# Endometrial ablation plus levonorgestrel releasing intrauterine system versus endometrial ablation alone in women with heavy menstrual bleeding: study protocol of a multicentre randomised controlled trial; MIRA2 trial

**DOI:** 10.1186/s12905-022-01843-6

**Published:** 2022-06-27

**Authors:** Tamara J. Oderkerk, Pleun Beelen, Peggy M. A. J. Geomini, Malou C. Herman, Jaklien C. Leemans, Ruben G. Duijnhoven, Judith E. Bosmans, Justine N. Pannekoek, Thomas J. Clark, Ben Willem J. Mol, Marlies Y. Bongers

**Affiliations:** 1 Department of Obstetrics and Gynaecology, Máxima Medical Centre, Postbox 7777, 5500 MB Veldhoven, The Netherlands; 2 Research School Grow, Maastricht University, Maastricht, The Netherlands; 3 Department of Obstetrics and Gynaecology, Jeroen Bosch Ziekenhuis, ‘s-Hertogenbosch, The Netherlands; 4 Clinical Trials Unit of the Netherlands Society of Obstetrics and Gynaecology, Department of Obstetrics and Gynaecology, Amsterdam University Medical Centres, Amsterdam, The Netherlands; 5 Department of Health Sciences, Faculty of Science, Vrije Universiteit Amsterdam, Amsterdam Public Health Research Institute, Amsterdam, The Netherlands; 6 University Department of Medicine and Obstetrics & Gynaecology, Birmingham Women’s Hospital, Birmingham, UK; 7 Department of Obstetrics and Gynaecology, Monash Medical Centre Clayton, Melbourne, Australia

**Keywords:** Heavy menstrual bleeding, Dysmenorrhoea, Endometrial ablation, Hysterectomy, Levonorgestrel-releasing intrauterine system, Pelvic pain

## Abstract

**Background:**

It is estimated that between 12 to 25% of women who undergo an endometrial ablation for heavy menstrual bleeding (HMB) are dissatisfied after two years because of recurrent menstrual bleeding and/or cyclical pelvic pain, with around 15% of these women ultimately having a hysterectomy. The insertion of a levonorgestrel-releasing intrauterine system (LNG-IUS) immediately after endometrial ablation may inactivate residual untreated endometrium and/or inhibit the regeneration of endometrial tissue. Furthermore, the LNG-IUS may prevent agglutination of the uterine walls preventing intrauterine adhesion formation associated with endometrial ablation. In these ways, insertion of an LNG-IUS immediately after endometrial ablation might prevent subsequent hysterectomies because of persisting uterine bleeding and cyclical pelvic pain or pain that arises de novo. Hence, we evaluate if the combination of endometrial ablation and an LNG-IUS is superior to endometrial ablation alone in terms of reducing subsequent rates of hysterectomy at two years following the initial ablative procedure.

**Methods/design:**

We perform a multicentre randomised controlled trial in 35 hospitals in the Netherlands. Women with heavy menstrual bleeding, who opt for treatment with endometrial ablation and without contraindication for an LNG-IUS are eligible. After informed consent, participants are randomly allocated to either endometrial ablation plus LNG-IUS or endometrial ablation alone. The primary outcome is the hysterectomy rate at 24 months following endometrial ablation. Secondary outcomes include women’s satisfaction, reinterventions, complications, side effects, menstrual bleeding patterns, quality of life, societal costs.

**Discussion:**

The results of this study will help clinicians inform women with HMB who opt for treatment with endometrial ablation about whether concomitant use of the LNG-IUS is beneficial for reducing the need for hysterectomy due to ongoing bleeding and/or pain symptoms.

*Trial registration* Dutch Trial registration: NL7817. Registered 20 June 2019, https://www.trialregister.nl/trial/7817.

**Supplementary Information:**

The online version contains supplementary material available at 10.1186/s12905-022-01843-6.

## Background

Heavy menstrual bleeding (HMB) is a common problem affecting one in four women between 30 and 50 years of age, with serious implications on quality of life and social functioning [1]. Endometrial ablation (EA) is a common minimally invasive, surgical treatment for women suffering from HMB. In the Netherlands, around 6000 EA-procedures are performed per year. EA is a uterus-preserving procedure that aims to destroy the endometrium, thus reducing or stopping menstrual bleeding [2]. However, approximately one in five women is dissatisfied after EA because of ongoing vaginal bleeding and/or dysmenorrhoea and seek further treatment [3–7]. A recent study by Beelen et al. showed that 20% of all women treated with EA received reinterventions and 10% ultimately underwent a hysterectomy [3]. Ongoing vaginal bleeding results from untreated or regenerated endometrium. Post-treatment dysmenorrhoea is likely the result of bleeding from areas of untreated or regenerated endometrium into a scarred and partially obliterated uterine cavity, causing a hematometra. Another cause is the presence of ectopic endometrial tissue within the underlying myometrium, known as adenomyosis [8–11].

Insertion of a levonorgestrel-releasing intrauterine system 52 mg (LNG-IUS, Mirena^®^, Bayer HealthCare Pharmaceuticals, Germany) immediately after EA may prevent persisting HMB and dysmenorrhoea. The local release of progesterone can potentially inactivate non-ablated or inhibit the regeneration of endometrial tissue as well as endometrial glands within the myometrium, thereby suppressing pelvic pain caused by adenomyosis [8, 12]. The LNG-IUS may also prevent intrauterine adhesion formation, which reduces the chance to develop hematometra. Uterine cavity obliteration developing in the period after EA mostly precludes later re-ablation or insertion of an LNG-IUS, often resulting in the preference for hysterectomy in case of discontent with the results of EA [9, 13].

A recently published systematic review concluded that combined EA + LNG-IUS treatment is a safe and promising procedure with a substantial lower reintervention and hysterectomy rate compared to treatment with EA alone [14]. A retrospective, single-centre cohort of 82 women in the study of Zhao et al. (2020) showed that women who received the combined treatment had a 0% hysterectomy rate compared to a 12% hysterectomy rate in women who received EA alone within a follow up period of 24 months [15]. In another retrospective single centre cohort of 88 women having EA combined with LNG-IUS, there was a 24% reduction in hysterectomy rate over a mean follow-up of 36 months [16]. Complication rates from combined treatment do not appear to be higher than treatment with EA alone [14].

Hence, in light of the potential benefits of combining EA and LNG-IUS and the availability of observational studies only [14], rigorous evaluation in a large multicentre randomised controlled trial (RCT) is essential. The MIRA2 trial will assess the effectiveness and cost-effectiveness of EA in combination with an LNG-IUS in comparison with EA alone in women with HMB using the rate of hysterectomy as the primary outcome measure.

## Methods/design

### Objective

The aim of this study is to assess whether the insertion of an LNG-IUS directly after EA is more effective in reducing the need for subsequent hysterectomy within two years of the EA in women with HMB and undergoing EA, as compared to EA alone. Also, the study will evaluate women’s satisfaction, reinterventions and complications, side effects, post-ablation menstrual bleeding patterns, quality of life, sick leave, and costs-effectiveness.

### Study design

The MIRA2 study is a nationwide multicentre randomised controlled clinical trial (RCT) in the Netherlands investigating the superiority of EA combined with concurrent LNG-IUS compared over EA alone. An economic evaluation will be performed alongside the trial. The study will be performed within the infrastructure of the Dutch Consortium for studies in Women’s Health. This study will be conducted in accordance with the Declaration of Helsinki as well as the Netherlands Medical Research Involving Human Subjects Act (WMO) and was approved as a primary review by the Máxima Medical Center medical ethics committee. Trial registration is available in the Dutch Trial Register (NL7817).

**Fig. 1:**
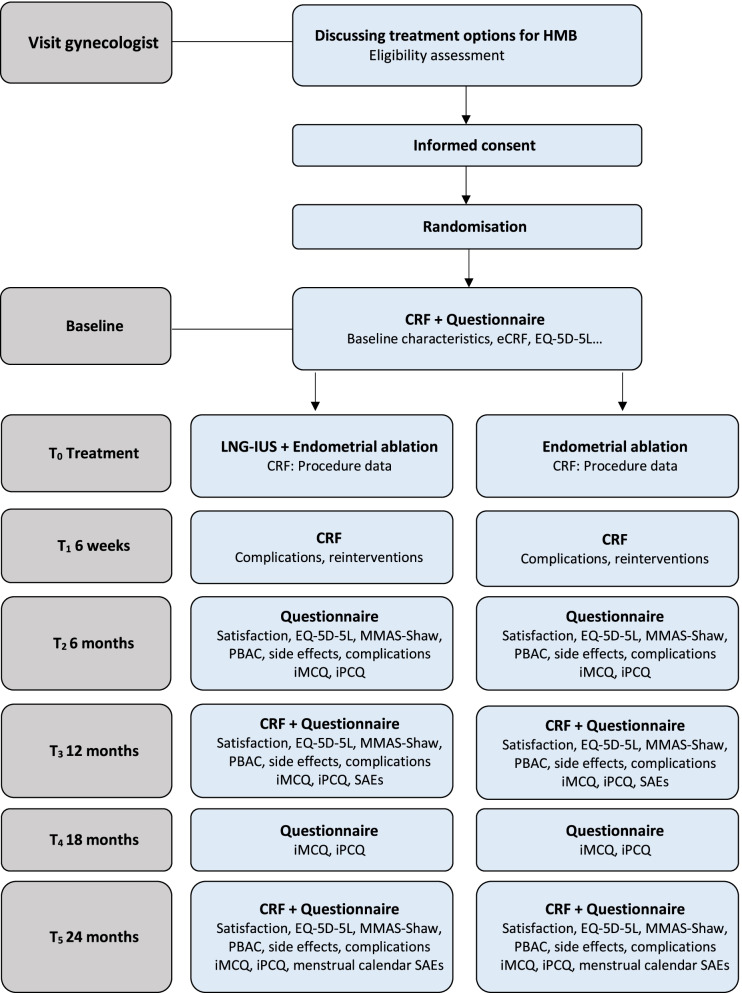
Flowchart of the MIRA2 trial. HMB = Heavy Menstrual Bleeding, CRF = Case Report Form, LNG-IUS = Levonorgestrel Intra-uterine system, EQ-5D-5L = The 5-level EuroQol version of quality-of-life questionnaire, MMAS-Shaw = menorrhagia multi-attribute scale questionnaire, PBAC = Pictorial Blood Loss Assessment Chart, iMCQ = iMTA Medical Consumption Questionnaire, iPCQ = iMTA Productivity Cost Questionnaire, SAE = Serious Adverse Event

A complete overview of the MIRA2 trial is presented in Fig. 1.

### Study population

Women suffering from HMB who opt for treatment with EA can participate in the trial. Women with contraindications for use of LNG-IUS are excluded from the trial. Other exclusion criteria are age of 60 years or older, suspicion of endometrial cancer and prior endometrial ablation. Moreover, women who are unable to read or speak Dutch or English to a level that allows them to fully understand the study description and informed consent cannot participate in the trial.

### Participants recruitment, randomisation and collection of data

Participants meeting the eligibility criteria will be counselled about the study by their physician or a research nurse, who will provide them with written information about the study. Women will be counselled according to the ‘shared decision making’ principle. Women who agree to participate in the study must provide written informed consent before randomization can take place by a research nurse or researcher.

Randomisation will be stratified by the presence of pre-existing dysmenorrhoea at baseline (pain during menstruation for which most of the menstrual periods pain medication is necessary) and by age (under and above 45 years old). Variable block sizes of size 2, 4 and 6 will be used.

The online software tool Castor EDC (www.castoredc.com) will be used for randomisation, sending questionnaires, and data collection. The computer will randomly assign a unique numeric code for every subject, consisting of the hospital and the consecutive participant number. A coded data set will be handled, and only the local investigator and the research nurse on site will have access to the key (code to personal information linkage). These coded data are available to investigators, research staff, and quality assurance and monitoring personnel. After the study is complete, data will be archived for a period of 15 years. Personal data is handled in compliance with the EU General Data Protection Regulation and the Dutch Act on Implementing the General Data Protection Regulation.

### Interventions

Hospitals using electrical, radiofrequency ablation (Novasure^®^, Hologic, Marlborough, MA, USA), the thermal balloon ablation (Thermablate^®^, Idoman Teoranta, Ireland and Librata^®^, Lina Medical, Denmark) or transcervical resection of the endometrium can participate in this trial. Hospitals performing this procedure must conduct a minimum of 15 procedures a year to assure quality and competency. The EA procedure may be performed under local anaesthesia in the outpatient office, under conscious sedation in an outpatient clinic or under general or spinal anaesthesia in an operating room, depending on the local conditions in the respective centre. In women allocated to EA + LNG-IUS, an LNG-IUS will be inserted immediately after finishing the EA procedure. The LNG-IUS can stay in place for a maximum of five years in case contraception is needed or longer in case contraception is not needed. Women allocated to the control group, only an EA will be performed.

### Outcome measures

The primary outcome will be the hysterectomy rate at two years after endometrial ablation as treatment for HMB. This will be measured using questionnaires in the follow-up period and checked by consulting the individual participant file. Secondary outcomes will include women’s satisfaction (on a 6-point Likert scale), condition-specific quality of life and general Quality of Life, severity of cyclical and non-cyclical pelvic pain, presence of (inter)menstrual bleeding, non-hysterectomy reintervention rates (removal LNG-IUS, medical or other surgical interventions), number and type of complications and adverse effects of treatment. Costs will be evaluated from a societal perspective. Outcomes will be collected via an online questionnaire completed by participants and via an online Case Report Form (CRF) completed by researchers.

#### Questionnaires for participants

Prior to treatment, participants will be asked to complete a baseline questionnaire containing a validated condition-specific quality of life measure Menorrhagia Multi-Attribute Scale (MMAS) as well as a validated EuroQol 5-level questionnaire [17–19]. The English language version of the MMAS questionnaire is shown in Additional file 1. Also, menstrual blood loss with a written Pictorial Blood Loss Assessment Chart (PBAC) score will be measured at baseline (Additional file 2) [20]. Participants will complete a questionnaire at six months follow-up asking about contraceptive use and smoking habits (Additional file 3). At 6-, 12- and 24-months participants will receive a questionnaire containing the MMAS questionnaire, the EQ-5D-5L questionnaire, and PBAC scoring, and questions on women’s satisfaction (6-point Likert scale), side effects of the LNG-IUS, and the presence of cyclic pelvic pain (pain score 0–10).

Societal costs (healthcare costs, lost productivity costs and informal care costs) are assessed using specifically adapted versions of the iMTA Productivity Cost Questionnaire (iPCQ) and the iMTA Medical Consumption Questionnaire (iMCQ) at 6, 12, 18 and 24 months (Additional file 4) [21, 22]. Participants will be asked to complete a menstrual calendar for one month to evaluate the intermenstrual bleeding 24 months after treatment (Additional file 5). An overview of questionnaires and timepoints is shown in Table 1.

**Table 1:** Schematic overview of follow up questionnaires for participants

Timepoints	T_0_: Baseline	T_2_: 6 months	T_3_: 12 months	T_4_: 18 months	T_5_: 24 months
1. 6 months follow up questionnaire		x			
2. Women’s satisfaction		x	x		x
3. EQ-5D-5L	x	x	x		x
4. PBAC	x	x	x		x
5. Cyclic pelvic pain and MMAS	x	x	x		x
6. Side-effects or complications		x	x		x
7. iMCQ and iPCQ		x	x	x	x
8. Menstrual calendar					x

EQ-5D-5L = The 5-level EuroQol version of quality-of-life questionnaire, PBAC = Pictorial Blood Loss Assessment Chart, MMAS = menorrhagia multi-attribute scale questionnaire, iMCQ = iMTA Medical Consumption Questionnaire, iPCQ = iMTA Productivity Cost Questionnaire

#### Electronic case report form for researchers

Baseline demographic characteristics, medical history, pre-existing dysmenorrhoea and current use of hormonal medication are recorded by the physician/research nurse in the electronic CRF. In addition, findings of the ultrasound examination will be collected in the eCRF, specifically, the existence of myomas (number, type and size) and presence of possible signs of adenomyosis.

At 6 weeks and 24 months after intervention the research nurse/physician will record post-treatment complications (expulsion or removal IUS, endometritis, uterine infection, bleeding (> 500 ml), uterine perforation caused by IUS insertion or EA) and reinterventions (including hysterectomy, removal of IUS, re-ablation or medical treatment).

## Statistical analysis

### Sample size

Based on a literature search of previous studies on hysterectomy rate after EA, we found variation in hysterectomy rates between 9 and 17.5% [3, 23–26]. An assessment in the Dutch population of hysterectomy rates after EA indicates a hysterectomy rate around 15% after previous EA in daily practice. For the sample size calculation, we assume a 15% hysterectomy rate after treatment with EA alone.

Based on a literature search on treatment with EA and LNG-IUS, we found two recently published studies with a comparable study population [15, 27]. These studies show hysterectomy rates after combined treatment between 0 and 11%. Therefore, in this study the hysterectomy rate in the group treated with EA and LNG-IUS is hypothesized to be 8% [15, 27]. Using an alpha of 0.05 and a beta of 0.2 and the 15% hysterectomy rate in the EA alone group and 8% in the EA + LNG-IUS group, 646 participants are needed to achieve 80% power. This enables the detection of a 7-percentage point’s difference between the group proportions. The sample size was calculated using the two-sided Z-Test with unpooled variance. Assuming a 10% loss-to-follow-up rate, we need to recruit 718 participants in the trial (359 per group). This sample size calculation was performed using PASS15.0.10.

### Data-analysis

The primary analysis will evaluate if treatment of HMB by combined EA and LNG-IUS is superior to treatment by EA alone. The primary outcome measure will be a relative risk with 95% confidence interval and chi-squared test for hysterectomy at 24 months after treatment. The null-hypothesis is that combined endometrial ablation (EA) and LNG-IUS does not decrease the hysterectomy rate. The primary outcome will be analysed on the intention-to-treat population. Interim analyses will not be conducted.

Quality of life and longitudinal data on PBAC-scores will be analysed using Generalized Estimating Equations with treatment group, time, and treatment group—time interaction as variables, and a random effect for participants. Secondary outcomes that are dichotomous will be expressed as percentages and relative risks along with corresponding 95% confidence intervals. Calculating P-values will be based on the chi-square test or Fisher exact test, whichever is applicable. For continuous variables, the means will be presented along with the standard deviation, or the medians will be presented along with the interquartile range. Differences between the treatment groups will be estimated as differences in means of medians with t-test or Mann–Whitney test for inference (e.g. NRS-score pain). Questionnaires will be processed with the appropriate algorithms and presented by the usual methods (EQ5D and MMAS), with the baseline values adjusted for. Continuous data from questionnaires will be transformed or categorised as appropriate for the distribution of the data.

Quality-of-life data will be modelled using techniques that allow for missing data. Additional interventions or reinterventions can be obtained from medical files and will therefore have low rates of missing data. For remaining secondary outcomes measured using questionnaires, multiple imputation will be considered if necessary.

In addition to the analysis of the primary outcome on the intention-to-treatment population, the risk for hysterectomy will also be estimated for the per-protocol population.

The per-protocol population will evaluate all participants who have completed their treatment as randomised. Those who are randomised to endometrial ablation have undergone this treatment. Those who are randomised to endometrial ablation together with the placement of an LNG-IUS have this treatment and the IUS remained in situ for at least 6 months. P-values < 0.05 will be considered to indicate statistical significance. The analysis will be done using the software Statistical Package for the Social Sciences (SPSS, Inc., Chicago, IL, USA).

### Subgroup analysis

Subgroup analyses will be conducted for women with pre-existing dysmenorrhoea, women with tubal ligation as contraception and for women under and over 45 years of age. Subgroup effects will be studied using interaction terms in a generalised linear model.

### Economic evaluation

A cost-effectiveness analysis will be conducted from a societal perspective over a 24-month time horizon. Costs and effects in the second year of follow-up will be discounted according to Dutch guidelines [28]. Quality-Adjusted Life-Years will be calculated based on the EQ-5D-5L using the Dutch EQ-5D-5L tariff [18]. A multiple imputation method developed by van Buuren et al. will be used to impute missing cost and effect data [29]. An estimation of cost and effect differences will be made using linear regression analyses, adjusted for confounders if necessary. To estimate statistical uncertainty surrounding the cost and effect differences, bias-corrected accelerated bootstrapping with 5,000 replications will be conducted. Calculating the incremental cost-effectiveness ratios (ICERs) requires dividing the cost difference between the groups by the difference in effects. To demonstrate the uncertainty around the ICER, boot-strapped cost-effect pairs will be plotted on cost-effectiveness planes. To estimate whether EA combined with an LNG-IUS is cost-effective in comparison to EA alone for various willingness-to-pay values (i.e. what society is willing to pay per additional unit of effect), cost-effectiveness acceptability curves will be estimated.

### Monitoring

Given that the study investigates an intervention within its indication of use interim analyses will not be conducted, and a Data Safety Monitoring Board will not be installed.

Monitoring will be performed by the Trialbureau Healthcare Evaluation which is affiliated to the NVOG Consortium, but fully independent from the sponsor. Based on the Study-Specific Monitoring program of the NVOG Consortium, site monitoring visits will be done to assess the performance of the participating sites and adherence to the study protocol. Qualified and independent monitors will have access to the data and source documents of the trial. Monitoring includes performance of one remote site start checklist and one on-site monitor visit per centre, and one remote close out checklist per centre.

### Harm

Adverse events (AE) are defined as specific undesirable experiences occurring to a subject during the study, which are defined as complications from the treatment. At 6 weeks, the following most common complications are assessed; expulsion or removal IUS, hysterectomy, endometritis, uterine infection, bleeding > 500 ml, uterine perforation caused by IUS insertion or endometrial ablation. Also, reinterventions will be assessed at 12 and 24 months in the CRF. All Serious Adverse Events (SAE) will be reported by the local investigator to the principal investigator. The principal investigator must report a SAE to an accredited METC within 15 days of receiving knowledge of it. For SAEs that result in death or are life-threatening, reporting will be expedited. An expedited report will be submitted not later than seven days after the investigator is informed of the adverse reaction.

## Discussion

Over the years, huge steps in uterus preserving treatments have been made for women with HMB, especially regarding the treatment with EA [2]. Still, a large group of women end up undergoing a hysterectomy to control symptoms of HMB or cyclical pelvic pain within 24 months of treatment with EA [3]. A hysterectomy is an expensive procedure associated with significant morbidity, arising from surgical and post-operative complications, as well as considerable psychological consequences [30, 31]. Additionally, women undergoing a hysterectomy have a median sick leave of 8.5 weeks, leading to substantial societal costs [30]. Preservation of the uterus is also an important reason for women to opt for a less invasive treatment, even if they know that there is a risk that such a less invasive treatment like EA could fail [31, 32].

Insertion of an LNG-IUS directly after EA might improve effectiveness of reducing HMB and dysmenorrhoea, while minimising costs to health care and society by significantly reducing the number of hysterectomies performed. We found no large prospective observational or randomised studies in the published medical literature evaluating combined treatment with EA and LNG-IUS. All seven observational studies described in the systematic review of Oderkerk et al. (2021) were of low methodological quality, mostly due to the use of retrospective designs and relatively small participant populations [14]. The MIRA2 trial is the first large multicentre randomised controlled trial comparing EA combined with LNG-IUS to EA alone. This trial will be conducted in a network of 35 hospitals in the Netherlands. The results of this study will be applicable to all women suffering from HMB who are considering treatment with endometrial ablation.

## Supplementary Information

**Additional file 1**. MMAS questionnaire. MMAS questionnaire English language version

**Additional file 2**. PBAC questionnaire. Written Pictorial Blood Loss Assessment Chart score English language version

**Additional file 3**. Six months follow up questionnaire. Six months follow-up questionnaire English language version

**Additional file 4**. iMCQ and iPCQ questionnaire. Adapted version of iMCQ and iPCQ questionnaires, English language version

**Additional file 5**. Menstrual calendar. Menstrual calendar, English language version

## Data Availability

The datasets from this study will be made available upon study completion and publication for the benefit/use of further research according to the Netherlands Organization for Health Research and Development (ZonMw) Grant Terms and Conditions, and in accordance with the FAIR standards (findable, accessible, interoperable and reusable). Reusing and sharing data will be covered in the informed consent procedure. The data collection system is hosted by the NVOG trial agency where research data can be saved and processed according to the FAIR principles. The datasets generated during and analysed during the current study are available in the Castor EDC repository, www.castoredc.com (v.8.21). The datasets used and/or analysed during the current study are available from the corresponding author on reasonable request.

## Abbreviations

HMB: Heavy menstrual bleeding
EA: Endometrial Ablation
LNG-IUS: Levonorgestrel releasing intrauterine system
RCT: Randomised controlled trial
eCRF: Electronic case report form
PBAC: Pictorial Blood Loss Assessment Chart
iPCQ: Productivity Cost Questionnaire
iMCQ: Medical Consumption Questionnaire
NVOG: Nederlandse vereniging obstetrie en gynaecologie (Dutch association for obstetrics and gynaecology)
