# Supplementary material for: Endometrial ablation plus levonorgestrel releasing intrauterine system versus endometrial ablation alone in women with heavy menstrual bleeding: study protocol of a multicentre randomised controlled trial; MIRA2 trial
Source: BMC Womens Health. 2022 Jun 27;22:257. doi: 10.1186/s12905-022-01843-6 (PMC9235075; doi:10.1186/s12905-022-01843-6)
Supplement: Supplementary file 1 — Additional file 1. MMAS questionnaire. MMAS questionnaire English language version [file 12905_2022_1843_MOESM1_ESM.pdf]

Case number:
Date:
Baseline 6 / 12 / 24 months follow-up

Shaw Questionnaire

In each of the questions below, choose the answer that most applies to you by checking the corresponding box. Please provide 1 answer per question.

1. Practical problems:

a. I have no practical problems, I do not menstruate more than I expect and I don't need to take extra precautions. ☐

b. I have to bring extra sanitary pads or tampons but no extra further precautions. ☐

c. I have to bring extra sanitary pads or tampons and clothing because of the chance of "leaking". ☐

d. I have serious problems with "leaking", I soil the bedding and need to have a toilet close by. ☐

2. Social life:

a. My social life is not affected during my period. I can enjoy life as usual. ☐

b. My social life is somewhat affected during my period. I sometimes have to change my plans. ☐

c. My social life is limited during my period, I rarely make plans. ☐

d. My social life disappears during my period, I am unable to plan something. ☐

3. Mental health:

a. During my period I have no worries and can handle it well. ☐

b. During my period I have some worries and fears. ☐

c. During my period I often feel sad and worry how to deal with it. ☐

d. During my period I feel depressed and I can't handle it. ☐

4. Physical health and well-being:

a. During my period I feel good and relaxed. I don't worry about my health. ☐

b. I usually feel good during my period. I am a bit worried about my health. ☐

c. During my period I often feel tired and not very well. I am worried about my health. ☐

d. During my period I feel very tired and not at all good. I am very worried about my health. ☐

Please check if you have answered all questions before continuing to the next page.

Case number:
Date:
Baseline 6 / 12 / 24 months follow-up

5. Work / daily activities:

a. My work / daily activities are not interrupted by my period. ☐
b. My work / daily activities are sometimes disturbed by my period. ☐
c. My work / daily activities are often disturbed by my period. ☐
d. My work / daily activities are severely disrupted by my period. ☐

6. Family life / relationships:

a. My family life / relationships are not influenced by my period. ☐
b. My family life / relationships suffer a little from my period. ☐
c. My family life / relationships suffer considerably under my period. ☐
d. My family life / relationships are seriously disturbed by my period. ☐

Please check whether you have filled in all the questions on <u>both pages.</u>
