# Supplementary material for: Endometrial ablation plus levonorgestrel releasing intrauterine system versus endometrial ablation alone in women with heavy menstrual bleeding: study protocol of a multicentre randomised controlled trial; MIRA2 trial
Source: BMC Womens Health. 2022 Jun 27;22:257. doi: 10.1186/s12905-022-01843-6 (PMC9235075; doi:10.1186/s12905-022-01843-6)
Supplement: Supplementary file 2 — Additional file 2. PBAC questionnaire. Written Pictorial Blood Loss Assessment Chart score English language version [file 12905_2022_1843_MOESM2_ESM.pdf]

Pictorial Blood Loss Assessment Chart

- How many days of blood loss did you have in the last month?

  □□□ [range 0-31]
- Number of complete soaked sanitary pads consumed in total in the last month?

  □□□ [range 0-100]
- Number of half-soaked pads used in total in the last month?

  □□□ [range 0-100]
- Number of sanitary pads with small drops of blood in total in the last month?

  □□□ [range 0-100]
- Number of full soaked tampons in total in the last month?

  □□□ [range 0-100]
- Number of half-soaked tampons in total in the last month?

  □□□ [range 0-100]
- Number of tampons with small drops of blood in the last month?

  □□□ [range 0-100]
