# Supplementary material for: Endometrial ablation plus levonorgestrel releasing intrauterine system versus endometrial ablation alone in women with heavy menstrual bleeding: study protocol of a multicentre randomised controlled trial; MIRA2 trial
Source: BMC Womens Health. 2022 Jun 27;22:257. doi: 10.1186/s12905-022-01843-6 (PMC9235075; doi:10.1186/s12905-022-01843-6)
Supplement: Supplementary file 3 — Additional file 3. Six months follow up questionnaire. Six months follow-up questionnaire English language version [file 12905_2022_1843_MOESM3_ESM.pdf]

Six months' follow-up questionnaire

Question 1.1: You are participating in the MIRA2 study and you have been assigned to either (1) a treatment with endometrial ablation or (2) a treatment with endometrial ablation in combination with insertion of a Mirena IUD. Was the treatment performed as decided by the randomisation?

□ no □ yes

If no: What treatment did you get?

□ Only Mirena IUD
□ Only an endometrial ablation
□ I did not get any treatment, because________
□ My uterus is removed, because __________(further to question 1.3)
□ Other, _________

Question 1.2: How do you prevent pregnancy from happening after treatment? (contraception)

□ I have a Mirena IUD
□ I had a sterilisation in history
□ I had a sterilisation the same time as my treatment
□ I use the pill, nuvaring, implanon or contraceptive injection
□ I use a condom
□ My partner is sterilized
□ Other,________________________________

Question 1.3: Do you smoke?

□ Yes, how many cigarettes a day? □□□ (range 1-100)

□ No
