# Supplementary material for: Endometrial ablation plus levonorgestrel releasing intrauterine system versus endometrial ablation alone in women with heavy menstrual bleeding: study protocol of a multicentre randomised controlled trial; MIRA2 trial
Source: BMC Womens Health. 2022 Jun 27;22:257. doi: 10.1186/s12905-022-01843-6 (PMC9235075; doi:10.1186/s12905-022-01843-6)
Supplement: Supplementary file 4 — Additional file 4. iMCQ and iPCQ questionnaire. Adapted version of iMCQ and iPCQ questionnaires, English language version [file 12905_2022_1843_MOESM4_ESM.pdf]

Research number:
Date:
6 months follow up

INSTRUCTIONS

Please read this first!

This questionnaire is about your health during the **last 6 months**.
The questionnaire consists of a number of parts.

1. The *first part* is about your work and the consequences for your work due to your health complaints (paid and unpaid work)
2. The *second part* concerns your use of (health) care.
3. The *third part* is about the medication u are using.

How long does it take to fill in the form?
It takes about 15 minutes to fill in the form.

How should you fill in the form?

1. Start with the first question and follow the numbering.
2. Put 1 x in the question box, unless the question states that you may put more than 1 x.
3. For some of the questions you may fill in a number or otherwise on the dotted line.
4. There are no wrong answers, it is also okay to give an estimate.

You cannot fill in the form on your own?
If you are unable to fill in the form on your own, maybe someone can help you, for example a member of the family. You can also contact the researcher of the MIRA2 study, to do the questionnaire by phone: Tamara Oderkerk, 040-8888380 or by email: MIRA2@zorgevaluatienederland.nl

Do you want to change an answer?
This is possible by clicking the buttons ‘return’ or ‘next’ to scroll through the questionnaire and to adapt your answers if necessary.

What happens to your answers?
Your answers will be used for research. Only the researchers will see your answers. That means therefore no one else.

Your general practitioner (GP) knows you participate in this study. Besides her/him, the researchers will not tell anyone that you have participated in this research project.

We greatly appreciate that you are willing to fill in this form for us!

Research number:
Date:
6 months follow up

Part 1. Your work and the consequences for your work due to your health complaints

1. Do you have a paying job?

☐ No, proceed to question 10

☐ Yes

2. How many hours a week do you paid work?

_________hour(s)

3. How many days a week do you do paid work?

_________day(s)

4. How often did you miss work in the last 6 months as a result of being sick?

☐ No, I didn't miss work the last 6 months

☐ Yes, sometimes

4.2.1 How often did you miss work the last six months? ________ time(s)
4.2.2 How many days did you miss work the last six months? ______ day(s)

☐ Yes, the entire 6 months

4.3.1 Since when did you miss work? __-__-___ (dd-mm-yyyy)

Sometimes, people with health complaints, need to call in sick. It could also be that someone does work, but performs less due to these health complaints.

5. Did you have physical problems during working days in the last 6 months?

☐ No, proceed to question 10

☐ Yes,

☐ a little

☐ a lot

6. How many days at work were you bothered by physical problems in the last 6 months?

_______ days

(You do not have to count the days that you were fully absent)

7. On the days that you were bothered by physical problems, was it perhaps difficult to get as much work finished as you normally do? On these days how much work could you do on average?

On these days I could not do anything I was able to do just as much as I normall

0 1 2 3 4 5 6 7 8 9 10

Research number:
Date:
6 months follow up

8. If you should catch up the work from the past 6 months that you were not be able to do work because of your health problems, how many hours would that be?

_______ hours

(You do not have to count the days that you were fully absent)

Explanation

Even for <u>unpaid work</u>, you can be bothered by physical problems. Sometimes as a result you might do less. For example you have trouble caring for your children or doing voluntary work. Or you are unable to pick up groceries, or to work in the garden. The following questions refer to this.

Were there any days you could do less <u>unpaid</u> work during the past 6 months, due to your physical problems?

☐ Yes

☐ No

If yes:

9. Which unpaid work couldn't you do completely or partly the past 6 months, due to complaints?

		No	Yes, how much?
1.	Work in and around the house		… hour(s), during … weeks
2.	Activities with your children		… hour(s), during … weeks
3.	Voluntary work		… hour(s), during … weeks
4.	Work for school/education		… hour(s), during … weeks

10. Did you have any help for unpaid work from someone you know for the last six months? (for example your partner, family member or neighbor. *If you have home care help: please fill in "no", this question will come back in part 2 "Health care use".*

☐ No, I didn't have any help

☐ Yes,

How many hours a week did you have help from someone you know?

_____hour(s) a week

During how many weeks did you have help from someone you know?

______week(s)

Research number:
Date:
6 months follow up

Part 2. Health care use

The next questions are about your health care use. We are interested in the amount of times or hours that you contacted health providers in **the past 6 months, for your own health**.

With 'contact' we mean consultation hours, appointments, phone appointments and house visits. Phone contact with the secretary or assistant to make the appointment, are not included.

11. In the past six months, were you in contact with…

		No	Yes, how many times?
A.	General practitioner		… times
B.	Social worker		… times
C.	Physiotherapist (of manual-, Caesar-, ergo-, Mensendieck-therapist)		… times
D.	Diet therapist		… times
E.	Homeopathic physician (including acupuncturist , osteopath)		… times
F.	Company doctor / Health and safety officer		… times
G.	Psychologist/psychotherapist/psychiatrist		… times
H.	Sexologist		… times
I.	Overig: …		… times

12. Have you received home care help in the last 6 months?

☐ No, I did not receive home care help.

☐ Yes:

		No	Yes, how much?
A.	Domestic help (house cleaning, groceries etc.		… hours each week, during … weeks
B.	Nurturing (getting dressed, showered etc.)		… hours each week, during … weeks
C.	Nursing (help with your medication, blood pressure measures etc.)		… hours each week, during … weeks

13. Did you receive hospital care in the past 6 months?

		No	Yes, how many times in the past 6 months?
A.	Emergency department		… times
B.	Ambulance transport to the hospital		… times
C.<br>D.	Outpatient clinic appointment with:<br>□ gynaecologist<br>□ other specialist *(total amount of outpatient clinic appointments with other specialists)*		 … times<br>… times

Research number:
Date:
6 months follow up

E.	Day treatment in the hospital:<br>*Day treatment 1 …*<br>*Day treatment 2 …*<br>*Day treatment 3 …*<br>*Day treatment 4 …*<br>*Day treatment 5 …*		 … times<br>… times<br>… times<br>… times<br>… times
F.	Surgical procedure (e.g. wound infection, bleeding)<br>*Procedure 1 …*<br>*Procedure 2 …*<br>*Procedure 3 …*<br>*Procedure 4 …*<br>*Procedure 5 …*		 <br><br>… times<br>… times<br>… times<br>… times<br>… times
G1.	Hospital admission (overnight)		… times, total of … days, of which:
G2.	Intensive care admission (overnight)		… days on the IC (total amount of days on the IC)
H.	Intake elsewhere (sleeping), e.g. GGZ (psychiatric department), revalidation centre etc.		… times, total of … days

14. In the past 6 months, did you visit other health providers or did other health providers visit you, that are not mentioned above?

☐ No

☐ Yes

a. If yes, which health care worker? …
b. How many times did you visit the health care worker/came the health care worker to you?… times

15. Have you undergone other treatments / admissions to hospital in the past 6 months that are not mentioned above?

☐ No

☐ Yes

c. If yes, what treatment or admission to the hospital, not mentioned above, did you get?
d. How many times have you been treated or administered? ______time(s)
e. How many days have you been treated or administered in total? ______day(s)

16. In the last 6 months, did you use medication?

☐ No, you are finished with this questionnaire.

☐ Yes

17. Which medication did you use extra in the last 6 months?

Research number:
Date:
6 months follow up

This is about medication you only use if you need them or if you want to. For example, if you have a lot of pain or if you cannot sleep.
Beneath you can see an example. If you use the medication mentioned in the example, please note this as well (with the amount that's applicable on your situation).
If you used more or less than what prescribed on the instruction, please fill in the real amount of medication you took.

**Medication name**	Dose	How many times a day	How many days in the past 6 months
paracetamol	*500 mg (1 tablet)*	*4 times*	*3 days*
Ibuprofen	*300mg*	*4 times*	*1 day*
Oxycodon	*5mg*	*6 times*	*2 days*
