# Supplementary material for: Endometrial ablation plus levonorgestrel releasing intrauterine system versus endometrial ablation alone in women with heavy menstrual bleeding: study protocol of a multicentre randomised controlled trial; MIRA2 trial
Source: BMC Womens Health. 2022 Jun 27;22:257. doi: 10.1186/s12905-022-01843-6 (PMC9235075; doi:10.1186/s12905-022-01843-6)
Supplement: Supplementary file 5 — Additional file 5. Menstrual calendar. Menstrual calendar, English language version [file 12905_2022_1843_MOESM5_ESM.pdf]

Menstrual calendar MIRA2 trial

Keep track of your blood loss this month in the calendar below. Please fill in for each cycle day in the coming month, including the days outside your period, whether you have had blood loss (and also the type of blood loss: brown, pink, dark red, bright red or clots), blood loss during intercourse and abdominal pain.
For example: cycle day 1 to day 6 menstruation, first 2 days with dark red bleeding, blood clots and abdominal pain, day 3 and 4 clear red blood loss, day 5 and 6 brown/pink blood loss. After that no more bleeding until day 24. On day 24 and 25 bleeding during intercourse with brown/pink discharge. No bleeding after that.

1st cycle day = first day of menstruation

Date 1st cycle day:
__-__-____

	**(Menstrual) Bleeding**					**Loss of blood during intercourse**	**Abdominal pain**
	Blood clots	Clear red	Deep red	Brown or pink	No bleeding		
1st cycle day							
2nd cycle day							
3rd cycle day							
4th cycle day							
5th cycle day							
6th cycle day							
7th cycle day							
8th cycle day							
9th cycle day							
10th cycle day							
11th cycle day							
12th cycle day							
13th cycle day							
14th cycle day							
15th cycle day							
16th cycle day							
17th cycle day							

18th cycle day							
19th cycle day							
20th cycle day							
21st cycle day							
22nd cycle day							
23rd cycle day							
24th cycle day							
25th cycle day							
26th cycle day							
27th cycle day							
28th cycle day							
29th cycle day							
30th cycle day							
31st cycle day							
